# Comparison of ultrasound risk stratification systems for pediatric thyroid nodules

**DOI:** 10.3389/fendo.2024.1350123

**Published:** 2024-03-20

**Authors:** Jing Yu, Yiyang Cui, Chao Fu, Xiao Ma, Caifeng Si, Yuanjing Huang, Kefei Cui, Yan Zhang

**Affiliations:** Department of Ultrasound, The First Affiliated Hospital of Zhengzhou University, Zhengzhou, China

**Keywords:** ultrasound (US), pediatric, thyroid nodule, fine needle aspiration (FNA), risk stratification system (RSS), Thyroid Imaging Reporting and Data System of the American College of Radiology (ACR-TIRADS), Chinese Thyroid Imaging Reporting and Data System (C-TIRADS), American Thyroid Association guidelines (ATA guidelines)

## Abstract

**Background:**

There is currently insufficient data to validate adult-based US risk stratification systems (RSSs) for the identification of malignant thyroid nodules in a pediatric population.

**Methods:**

From October 2016 and May 2023, 173 thyroid nodules of pediatric patients (age ≤ 18 years) with definitive pathology results and ultrasound (US) examination within 1 month before surgery or fine-needle aspiration (FNA) biopsy in our institution were enrolled in this study. The clinical and US characteristics of these nodules were retrospectively reviewed and categorized according to the ACR-TIRADS, C-TIRADS, and ATA guidelines. The diagnostic performance of US-based FNA criteria (original and simulating) of the three guidelines in thyroid cancer detection was estimated.

**Results:**

The three RSSs had similar AUC according to the categories(0.849-0.852, all *P* > 0.05). When combined with the original FNA criteria of the three RSSs to manage the nodules, the FNA rate of ACR-TIRADS and C-TIRADS were significantly less than ATA guidelines (53.18% vs. 64.63%, *P* < 0.05, and 52.60% vs. 64.63%, *P* < 0.05). The missed malignancy rate (MMR) and unnecessary FNA rate (UFR) of ATA guidelines (50.00%, 35.85%) was highest among the three RSSs, followed by the C-TIRADS (37.80%, 19.57%) and the ACR-TIRADS (37.04%, 19.57%). When nodules < 1 cm with the highest category in each RSS biopsied, that is when using the simulating FNA thresholds, the MMR was reduced overall (all *P* < 0.001), without a change in the UFR (all *P* > 0.05). All the three RSSs showed a substantial improvement in accuracy and malignant detection rate (all *P* < 0.05).

**Conclusion:**

The ACR-TIRADS, C-TIRADS, and ATA guidelines showed high missed malignancy rates when using their original recommended FNA criteria. When nodules < 1 cm with the highest category in each RSS biopsied, the missed malignancy rate of each RSS was decreased. Decreasing the FNA thresholds for highly suspicious malignant nodules may therefore be an effective means of managing malignant thyroid nodules in pediatric patients.

## 1 Introduction

Ultrasound plays a pivotal role in identifying thyroid nodules for diagnostic purposes. In adults, the detection rate ranges from 10% to 70%, with a malignancy rate of approximately 5% to 10% (1). Conversely, children and adolescents exhibit a lower thyroid nodule detection rate (about 0.5% to 1.6%), yet their malignancy risk is considerably higher compared to adults, ranging from 25% to 30%. Moreover, malignant thyroid nodules in the pediatric population are more prone to lung metastases, extrathyroidal growth, and lymph node metastases than in adults (2–4). Hence, achieving accurate preoperative differentiation between benign and malignant thyroid nodules in children is essential for effective nodule management and minimizing surgical risks.

Given the typically indolent nature of thyroid malignancies, various US-based risk stratification systems (RSSs) have been established to optimize the balance between minimizing unnecessary biopsies for benign nodules and ensuring accurate identification of malignant nodules (5–11). hree widely utilized systems include the American Thyroid Association (ATA) guidelines from 2015 (9), the American College of Radiology’s Thyroid Imaging Reporting and Data System (ACR-TIRADS) introduced in 2017 (7), and the Chinese TIRADS (C-TIRADS) from the Superficial Organ and Vascular Ultrasound Group of the Society of Ultrasound in Medicine of the Chinese Medical Association in 2020 (11). The majority of current RSSs are adults-based and several studies have shown the diagnostic utility of the above three RSSs in the management of thyroid nodules in adults (12, 13). While these RSSs are extensively applied in managing thyroid nodules in adults, their utility in pediatric cases remains less explored, with varying results in existing studies (14–16). It is noteworthy that pediatric thyroid volumes are smaller than those in adults, and biological differences in the behavior of thyroid malignancies between children and adults exist. Additionally, the effects of aging bring about changes that may impact the applicability of RSSs across different age groups. Concerns arise regarding the potential disparity in RSS application between children and adults, as well as the appropriateness of using nodule biopsy cutoff sizes established for adults in pediatric patients. This study aims to assess and compare the diagnostic performance of three adult-based RSSs (ACR-TIRADS, C-TIRADS, and ATA guidelines) for detecting thyroid malignancy via ultrasound in pediatric thyroid nodules.

## 2 Materials and methods

The research and clinical trials ethics committee of The First Affiliated Hospital of Zhengzhou University in China has granted approval for this single-center retrospective study (approval number 2022-KY-0974-001). Given the retrospective nature of the study, written informed consent was waived as it involves the analysis of existing data.

### 2.1 Patients

We conducted an examination of all thyroid nodules in individuals aged 18 or below who underwent thyroidectomy or fine-needle aspiration (FNA) biopsy, coupled with US evaluations, at our institution from October 2016 to May 2023. This assessment was based on databases containing consecutive records of pediatric thyroid biopsy and surgery outcomes. Inclusion criteria comprised: (I) a thyroid US examination conducted within one month before surgery or FNA; (II) having undergone surgery or FNA biopsy; and (III) possessing definitive histopathological or cytological results for the target nodules. Exclusion criteria included: (I) target nodules with indefinite histopathological or cytological results; (II) incomplete ultrasound image data; and (III) patients who had undergone chemotherapy or radiotherapy. Ultimately, our study encompassed a total of 173 thyroid nodules observed in 152 patients.

### 2.2 US examinations and analysis of US images

We conducted ultrasound examinations using a 5–14-MHz or 10–12-MHz linear probe and real-time ultrasound systems (EPIQ7C and EPIQ 5, Philips Healthcare; AplioXG and Aplio500, Toshiba Medical Systems; Mindray Resona 7T, Mindray Medical Systems). US examinations were performed by two radiologists with more than 10 years of clinical experience performing thyroid US. Nodule images were consistently obtained with at least one grayscale image in both transverse and longitudinal planes during the US examination. Additional images were captured to highlight the important ultrasound characteristics of the nodules. Subsequently, the US features of each nodule were analyzed by a senior radiologist with 34 years of clinical experience in thyroid imaging. The US features were included composition (mixed/cystic or purely cystic/solid or purely solid), echogenicity (isoechoic/hyperechoic/anechoic/hypoechoic), shape (wider-than-taller/taller-than-wider), margin (smooth/ill-defined/irregular or lobulated/extrathyroidal), and echogenic foci (macrocalcifications/none/large comet-tail artifacts/microcalcifications). Finally, another radiologist who deep-learned ACR-RADS, C-TIRADS, and ATA guidelines classified the nodules according to the three RSS. All radiologists involved in US examination, US imaging analysis or nodule classification were blinded to the FNA outcomes and the ultimate diagnoses of the nodules.

In the ACR-TIRADS (7), suspicious ultrasound features such as solid or almost completely solid composition, hypoechoic/very hypoechoic texture, taller-than-wide shape, lobulated or irregular/extra-thyroidal extension, and punctate echogenic foci were considered. These features were assigned 2 points, 2 points/3 points, 3 points, 2 points/3 points, and 3 points, respectively. In the C-TIRADS (11), features like solid component, marked hypoechoic texture, irregular margins/ill-defined or extrathyroidal extension, microcalcifications, and vertical orientation were considered suspicious, each carrying equal weight (one point). The cumulative numerical score determined the final category in ACR-TIRADS and C-TIRADS. In the ATA guidelines (9), nodules were directly assigned to different categories based on their ultrasound features. For instance, a solid hypoechoic nodule or almost completely hypoechoic nodule with certain characteristics, such as microcalcifications, taller-than-wide shape, irregular margins, rim calcifications with a small extrusive soft tissue component, or evidence of extrathyroidal extension, was classified as high suspicion category.

### 2.3 FNA thresholds of the three RSSs

ACR-TIRADS, C-TIRADS, and ATA guidelines have all advocated for an increased recommendation of FNA to manage thyroid nodules, aiming to minimize unnecessary biopsies of benign nodules while ensuring a higher proportion of biopsies for malignant nodules. These RSSs establish specific size thresholds for each nodule classification, advising FNA when the size exceeds these thresholds and refraining from recommending FNA if the nodule falls below the specified size. The original FNA thresholds of the three guidelines are summarized in **Table 1**.

**Table 1:** The original and simulating FNA thresholds of the ACR-TIRADS, C-TIRADS, and ATA guidelines.

Guideline	Score or nodule characteristics	FNA thresholds	
		Original	Simulating
ACR-TIRADS			
TR1	0 point (Benign)	No FNA	No FNA
TR2	2 points (Not suspicious)	No FNA	No FNA
TR3	3 points (Mildly suspicious)	≥25mm	≥25mm
TR4	4 - 6 points (Moderately suspicious)	≥15mm	≥15mm
TR5	≥ 7 points (Highly suspicious)	≥10mm	0
C-TIRADS			
TR2	-1 point (Benign)	No FNA	No FNA
TR3	0 point (Probably benign)	No FNA	No FNA
TR4A	1 points (Mildly suspicious)	>15mm	>15mm
TR4B	2 points (Moderately suspicious)	>10mm	>10mm
TR4C	3 - 4 points (Highly suspicious)	>10mm	0
ATA guidelines			
TR1	Benign	No FNA	No FNA
TR2	Very low suspicion	No FNA	No FNA
TR3	Low suspicion	>15mm	>15mm
TR4	Intermediate suspicion	>10mm	>10mm
TR5	High suspicion	>10mm	0

Given the smaller thyroid volumes in children compared to adults, age-related changes, and distinct biological behaviors of thyroid cancers in pediatric and adult populations, there is a concern that RSSs might be applied differently for thyroid nodules in these two groups. Furthermore, the biopsy cutoff sizes established for adults may not be suitable for pediatric patients. Consequently, we adjusted the FNA thresholds for the highest category (referred to here as simulating FNA thresholds) to assess the impact of nodule size cutoffs on diagnostic accuracy. In this simulated scenario, all nodules classified as ACR-TIRADS 5, C-TIRADS 4C, and ATA guidelines 5 were recommended for FNA. The simulating FNA thresholds of the three guidelines are also summarized in **Table 1**.

### 2.4 Statistical analysis

Our main focus was to assess the effectiveness of ACR-TIRADS, C-TIRADS, and ATA guideline biopsy criteria in identifying malignant nodules in children. To do this, we calculated various diagnostic performance metrics including sensitivity, specificity, accuracy, FNA rate (the number of nodules recommended for FNA among all nodules), unnecessary FNA rate (UFR, the number of benign nodules among those recommended for biopsy), missed malignancy rate (MMR, the proportion of malignant nodules among those not recommended for biopsy), and malignant detection rate (MDR, the proportion of malignant nodules recommended for biopsy among all nodules). Statistical comparisons of these diagnostic indicators were conducted using the McNemar test or Pearson test. Demographic characteristics between benign and malignant nodules, such as nodule size and patient age, were compared using the Mann–Whitney U test for continuous data. Additionally, the area under the receiver operating characteristic curves (AUCs) was calculated and compared using the Z-test or the DeLong test. Statistical analyses were performed using MedCalc 18.2.1 and SPSS 26.0 software, with significance set at a two-sided *P* < 0.05.

## 3 Results

### 3.1 Patient and nodule characteristics

In this study, a total of 173 thyroid nodules from 152 patients were examined. Among these patients, 107 were female (70.4%), and 45 were male (29.6%). The median age of the participants was 16.00 (13.00, 17.00) years (with a range of 2-18 years). There were no significant differences in terms of sex or age between children with malignant nodules and those with benign nodules. Of the 173 thyroid nodules, pathologic findings of 34 (19.65%) nodules were obtained by FNA, and pathologic results of 139 (80.35%) nodules were obtained surgically. In the 34 nodules obtained by FNA, 32 (94.12%) were benign and 2 (5.88%) were malignant. In the 139 nodules obtained surgically, 37 (26.62%) were benign and 102 (73.38%) were malignant. Finally, 69 (39.9%) nodules were identified as benign, while 104 (60.1%) nodules were classified as malignant. The FNA cytopathology results of nodules were diagnosed based on the 2017 version of the Bethesda system for reporting thyroid cytopathology (17). The nodules were diagnosed as benign when the FNA results were Bethesda II and were diagnosed as malignant when the FNA results were Bethesda VI. The nodules with Bethesda I, III, IV, and V were excluded from our study. The 5th series of the WHO Classification of Thyroid Tumors were used to diagnosis surgical pathology results of nodules (18). A significant number of malignant nodules were identified as papillary thyroid carcinomas (86 cases), while 15 cases were classified as follicular carcinomas, 2 as medullary carcinomas, and 1 as rhabdomyosarcomatosum. Among benign nodules, nodular goiters were the predominant type (56 cases), followed by 9 cases of inflammatory lesions, 2 cases of follicular adenomas, and 2 cases of simple goiters. The median size of all included thyroid nodules was 24.58 mm (9.00, 47.60), with no statistically significant difference observed in sizes between benign and malignant nodules (26.50 (9.00, 39.75) mm vs. 23.31 (8.7, 34.87) mm, *P* > 0.05). Further details about patient characteristics and nodule features are provided in **Table 2**. Distinct US characteristics were observed between benign and malignant nodules. Malignant nodules exhibited features such as being solid or purely solid, hypoechoic, taller-than-wider, ill-defined, irregular or lobulated, showing extrathyroidal extension, and microcalcifications (all *P* < 0.05). In contrast, benign nodules tended to be mixed, cystic or purely cystic, isoechoic, hyperechoic, anechoic, wider-than-taller, smooth, with macrocalcifications, and no echogenic foci or only large comet-tail artifacts (all *P* < 0.05). These findings are summarized in **Table 2**, outlining both the basic patient characteristics and the ultrasound features of the thyroid nodules.

**Table 2:** Clinical characteristics and US features.

Basic characteristics	Total	Benign	Malignant	*P* value
Patient	152 (100.0)	54 (35.53)	98 (64.57)	–
Sex				0.996
Female	45 (29.61)	16 (29.63)	29 (29.59)	
Male	107 (70.49)	38 (70.37)	69 (70.41)	
Age (years)*	16.00 (13.00,17.00)	15.00 (12.00,17.00)	16 .00 (13.00,18.00)	0.304
Nodule	173 (100.0)	69 (39.88)	104 (60.12)	
Median size (mm)*	24.58 (9.00, 47.60)	26.50 (9.00, 39.75)	23.31 (8.7, 34.87)	0.062
Size range (mm)	3 - 74	3 - 67	3.8 - 74	
Group				0.929
<10 mm	47 (27.17)	19 (27.54)	28 (26.92)	
≥10 mm	126 (72.83)	50 (72.46)	76 (73.08)	
Composition				0.000
Solid or purely solid	133 (76.88)	32 (46.38)	101 (97.12)	
Mixed	37 (21.39)	34 (49.27)	3 (2.88)	
Cystic or purely cystic	3 (1.73)	3 (4.35)	0 (0)	
Echogenicity				0.000
Isoechoic	50 (28.90)	34 (49.27)	16 (15.38)	
Hypoechoic	111 (64.16)	23 (33.33)	88 (84.62)	
Hyperechoic	9 (5.20)	9 (13.04)	0 (0)	
Anechoic	3 (1.73)	3 (4.35)	0 (0)	
Shape				0.001
Taller-than-wider	27 (15.61)	3 (4.35)	24 (23.08)	
Wider-than-taller	146 (84.39)	66 (95.65)	80 (76.92)	
Margin				0.000
Smooth	67 (38.73)	51 (73.91)	16 (15.38)	
Ill-defined	41 (23.70)	13 (18.84)	28 (26.92)	
Irregular or lobulated	57 (32.95)	5 (7.25)	52 (50.00)	
Extrathyroidal extension	8 (4.62)	0 (0)	8 (7.69)	
Echogenic foci				0.000
Microcalcifications	92 (53.18)	12 (17.39)	80 (76.92)	
Macrocalcifications	5 (2.89)	4 (5.80)	1 (0.96)	
None	64 (36.99)	41 (59.42)	23 (22.12)	
Large comet-tail artifacts	12 (6.94)	12 (17.39)	0 (0)	

*Data in parentheses are percentages the interquartile range i. e. 25% and 75%. Data in parentheses are percentages unless otherwise indicated.

### 3.2 Multivariate logistic regression analysis for malignant pediatric thyroid nodules

Five variables with statistical significance in the univariate analysis were included in the multivariate logistic regression analysis as follows: composition (0 for mixed/cystic or purely cystic and 1 for solid or purely solid), echogenicity (0 for isoechoic/hyperechoic/anechoic and 1 for hypoechoic), shape (0 for wider-than-taller and 1 for taller-than-wider), margin (0 for smooth/ill-defined and 1 for irregular or lobulated/extrathyroidal), and echogenic foci (0 for macrocalcifications/none/large comet-tail artifacts and 1 for microcalcifications). Taking pediatric thyroid carcinoma as the dependent variable, the 5 preoperative factors with statistical significance were used in the regression model as independent variables. The results showed that composition [odds ratio (OR): 12.411, 95% CI: 2.873-54.290; P=0.001], margin (OR: 6.266, 95% CI: 2.078-18.892; P=0.001), and echogenic foci (OR: 4.150, 95% CI: 1.571-11.355; P=0.006) were independent risk factors for pediatric thyroid carcinoma (see **Table 3** for details). The echogenicity (OR: 1.194, 95% CI: 0.392-3.639; P=0.755) and shape (OR: 1.660, 95% CI: 0.402-6.851; P=0.483) were not the independent risk factors for pediatric thyroid carcinoma.

**Table 3:** Multivariate Logistic regression analysis for malignant pediatric thyroid nodules.

Covariates	*P* value	OR (95% CI)
Composition		
mixed/cystic or purely cystic		
solid or purely solid	0.001	12.411 (2.873-54.290)
Echogenicity		
isoechoic/hyperechoic/anechoic		
hypoechoic	0.755	1.194 (0.392-3.639)
Shape		
wider-than-taller		
taller-than-wider	0.483	1.660 (0.402-6.851)
Margin		
smooth/ ill-defined		
irregular or lobulated/extrathyroidal	0.001	6.266 (2.078-18.892)
Echogenic foci		
macrocalcifications/none/large comet-tail artifacts		
microcalcifications	0.006	4.150 (1.571-11.355)

### 3.3 Comparison of diagnostic performance of the three RSSs according to FNA recommendations

**Figure 1:**
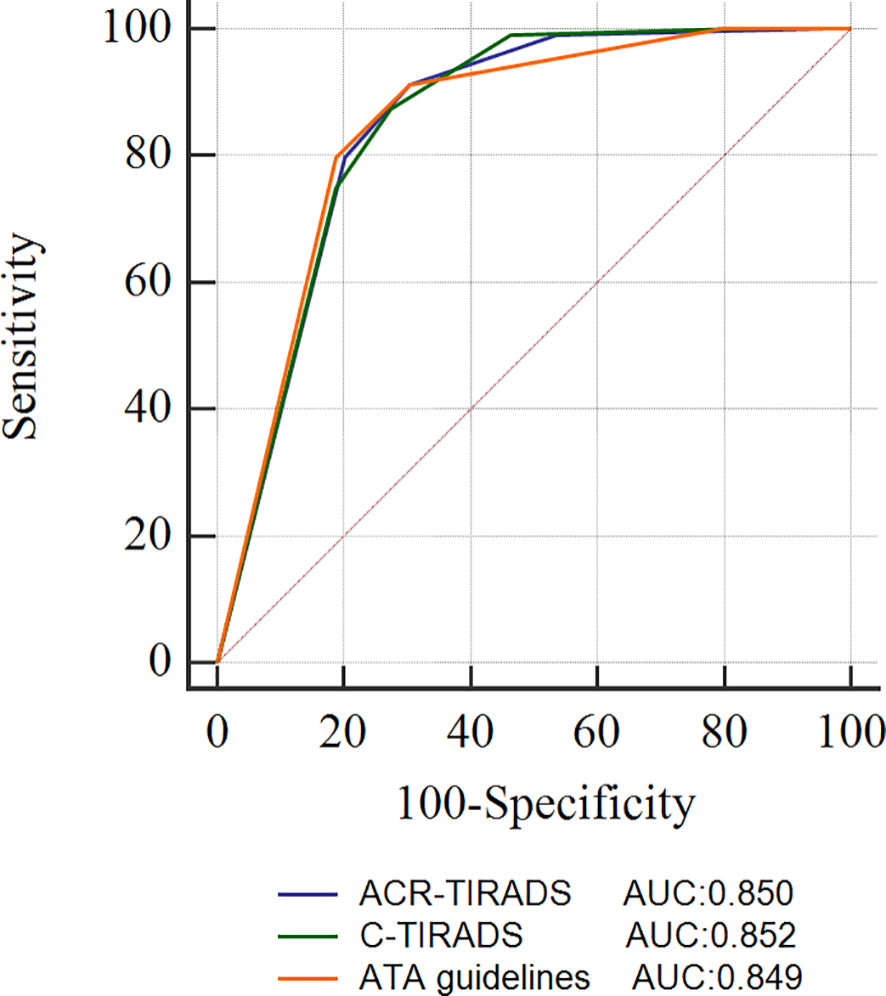
ROC curves of the ACR-TIRADS, C-TIRADS, and ATA guidelines. ACR-TIRADS, Thyroid Imaging Reporting and Data System of the American College of Radiology; C-TIRADS, Chinese Thyroid Imaging Reporting and Data System; ATA, American Thyroid Association guidelines; ROC, receiver operating characteristic; AUC, area under the ROC curve.

All thyroid nodules underwent classification according to both ACR-TIRADS and C-TIRADS. However, nine nodules could not be classified using the ATA guidelines, and of these, 7 (77.78%) were found to be malignant. The ROC curve, displayed in **Figure 1**, illustrates the diagnostic performance of the three RSSs. The cutoff category for distinguishing malignant from benign nodules was determined as 4 for ACR-TIRADS, 4B for C-TIRADS, and high suspicion for ATA guidelines based on the ROC curve. Consequently, nodules were categorized as benign if classified as TR1-TR3 in ACR-TIRADS, TR2-TR4A in C-TIRADS, and benign to intermediate suspicion in ATA guidelines. Nodules were considered malignant if classified as TR4 to TR5 in ACR-TIRADS, TR4B-TR5 in C-TIRADS, and high suspicion in ATA guidelines. Using these criteria, the AUCs (95% confidence interval) of ACR-TIRADS, C-TIRADS, and ATA guideline were 0.850 (0.788-0.900), 0.852 (0.790-0.901), and 0.849 (0.786-0.899), respectively. All the three RSSs demonstrated similar AUCs (all *P* > 0.05). When incorporating FNA criteria from the three RSSs to manage nodules, ACR-TIRADS and C-TIRADS recommended FNA for 92 and 91 nodules, respectively. Both were significantly fewer than the recommendations from ATA guidelines (53.18% vs. 64.63%, P < 0.05, and 52.60% vs. 64.63%, *P* < 0.05). Sensitivity and MDR did not significantly differ among the three RSSs (all *P* > 0.05). ACR-TIRADS and C-TIRADS exhibited higher specificity and accuracy compared to ATA guidelines (73.91% vs. 43.28%, 73.91% vs. 43.28%, *P* < 0.001; and 72.25% vs. 59.19%, 71.68% vs. 59.19%, *P* < 0.05). UFR based on ATA guidelines was significantly higher than that based on ACR-TIRADS and C-TIRADS (35.85% vs. 19.57%, *P* < 0.05; 35.85% vs. 19.57%, *P* < 0.05). The MMR was highest for ATA guidelines (50.00%), followed by C-TIRADS (37.80%) and ACR-TIRADS (37.04%). These results are summarized in **Table 4**.

**Table 4:** Diagnostic performance of the ACR-TIRADS, C-TIRADS, and ATA guidelines according to their original FNA thresholds.

	ACR-TIRADS	C-TIRADS	ATA guidelines	*P* value		
				ACR-TIRADS vs. C-TIRADS	ACR-TIRADS vs. ATA guidelines	C-TIRADS vs. ATA guidelines
Nodules recommended for FNA	92	91	106	–	–	–
Benign nodules in FNA	18	18	38	–	–	–
Malignant nodules in FNA	74	73	68	–	–	–
Sensitivity	71.15	70.19	70.10	0.879	0.870	0.989
Specificity	73.91	73.91	43.28	1.000	0.000	0.000
Accuracy	72.25	71.68	59.15	0.905	0.011	0.016
FNA rate	53.18	52.60	64.63	0.914	0.033	0.025
Unnecessary FNA rate	19.57	19.57	35.85	0.971	0.011	0.013
Missed malignancy rate	37.04	37.80	50.00	0.919	0.127	0.151
Malignant detection rate	42.77	42.20	41.46	0.913	0.808	0.892

### 3.4 Comparison of diagnostic performance of the three RSSs according to simulating FNA threshold

The malignancy rates of ACR TR5, C TR4C, and ATA TR5 were 85.57% - 86.46%. Take ACR-TIRADS for example, there were 35 nodules with a size of <10mm in TR5. And the malignancy rates were 74.29%. Additionally, 65.38% had lymph node metastases in the malignant nodules sized <10mm of ACR TR5. Therefore, the malignant nodules less than 10mm should not be easily ignored in pediatric thyroid nodules. Given this, we set the simulating FNA threshold to 0 in the highest category in each RSS. When biopsying nodules < 1 cm with the highest category in each RSS, using the simulated FNA thresholds significantly enhanced the overall sensitivity of each RSS (all *P* < 0.001), albeit at the cost of reduced specificity (all *P* > 0.05). Furthermore, the overall MMR decreased significantly (all *P* < 0.001), while the UFR remained unchanged (all *P* > 0.05). Specifically, the MMR of the ATA guidelines dropped from 50.00% to 0, and the UFR decreased from 35.85% to 32.64%. All three RSSs exhibited notable improvements in accuracy and MDR (all *P* < 0.05). These results are summarized in **Table 5**.

**Table 5:** Diagnostic performance of the ACR-TIRADS, C-TIRADS, and ATA guidelines according to the simulating FNA thresholds compared to the original FNA thresholds.

	ACR-TIRADS			C-TIRADS			ATA guidelines		
	original	Simulating	*P* value	original	Simulating	*P* value	original	Simulating	*P* value
Nodules recommended for FNA	92	127	–	91	126	–	106	144	–
Benign nodules in FNA	18	27	–	18	26	–	38	47	–
Malignant nodules in FNA	74	100	–	73	100	–	68	97	–
Sensitivity	71.15	96.15	0.000	70.19	96.15	0.000	70.10	100.00	0.000
Specificity	73.91	60.87	0.102	73.91	62.32	0.144	43.28	29.85	0.106
Accuracy	72.25	82.08	0.029	71.68	82.66	0.015	59.15	71.34	0.020
Unnecessary FNA rate	19.57	21.26	0.759	19.57	20.63	0.877	35.85	32.64	0.596
Missed malignancy rate	37.04	8.70	0.001	37.80	8.51	0.000	50.00	0	0.000
Malignant detection rate	42.77	57.80	0.005	42.20	57.80	0.004	41.46	59.14	0.001

## 4 Discussion

The existing literature lacks sufficient evidence supporting the utilization of adult-based US-based RSSs for distinguishing between malignant and benign thyroid nodules in pediatric patients. In our study, we applied three adult-based RSSs to pediatric patients and found that the diagnostic performance in the pediatric population was comparable to that observed in adults (13, 19). Specifically, the ATA guidelines exhibited lower accuracy and higher unnecessary FNA rates compared to ACR-TIRADS and C-TIRADS based on FNA recommendations. Each guideline demonstrated a high MMR ranging from 37.04% to 50.00% when employing their original FNA criteria. However, when simulating FNA thresholds were utilized, the overall MMR for each RSS decreased (0-8.70%) without affecting the unnecessary FNA rate. Notably, sensitivity, accuracy, and malignant detection rate were significantly improved. Consequently, our findings suggest that applying adult-based thyroid ultrasound RSSs in pediatric populations could be beneficial, and that biopsying the highest category nodules smaller than 1 cm could enhance diagnostic performance.

In alignment with previous research findings, our study identified several US characteristics indicative of high and low risk in pediatric thyroid nodules. Malignant thyroid nodules exhibiting mixed composition, isoechoic/hyperechoic features, wider-than-taller shapes, and macrocalcifications had a lower occurrence compared to benign thyroid nodules with these ultrasound features, consistent with findings from prior adult-based studies (20). Conversely, malignant thyroid nodules sharing similar ultrasound features had a higher incidence compared to benign thyroid nodules with solid or purely solid composition, hypoechoic features, taller-than-wider shapes, irregular or lobulated/extrathyroidal extension, and microcalcifications. These observations were also in line with earlier adult-based studies (20). Notably, we observed that taller-than-wider shapes exhibited high specificity (95.65%) but an exceptionally low sensitivity of 23.08% in pediatric patients, significantly lower than its sensitivity in adult thyroid cancer patients (21). The incidence of taller-than-wider shapes in our study was only 15.61% (27/173), consistent with a meta-analysis by Al Nofal et al., which indicated the rarity of taller-than-wider shapes in children and adolescents with thyroid nodules (22). We speculate that the lower occurrence of taller-than-wider shapes in children and adolescents may be attributed to their smaller thyroid volumes and limited tumor growth compared to adults. Additionally, it has been proposed that the proportion of microscopic thyroid cancer in children and adolescents is substantially lower than in adults, and later stages of thyroid cancers in this age group often present with irregular shapes (23). Consequently, the diagnostic value of taller-than-wider shapes in pediatric patients with thyroid cancer is reported to be lower than in adult patients. Future risk stratification systems may need to reevaluate the diagnostic performance of taller-than-wider shapes in malignant nodules in pediatric patients.

Our study demonstrated that ACR-TIRADS, C-TIRADS, and ATA guidelines exhibit high overall diagnostic efficacy for thyroid nodules in pediatric patients, with AUCs ranging from 0.850 to 0.855. Consistent with our findings, a recent study by Uner et al. (15) reported that ACR TI-RADS achieved an AUC of 0.890 for diagnosing thyroid nodules in children and adolescents. All three RSSs recommended FNA management for thyroid nodules based on ultrasound signs and nodule size, aiming to reduce unnecessary FNA rates for benign nodules and enhance the detection of malignant nodules. Previous studies in adults have indicated that ACR TI-RADS has a lower rate of unnecessary FNA compared to other RSSs (24–27), potentially attributed to its larger suggested FNA thresholds. However, it’s worth noting that ACR TI-RADS has been associated with a higher missed malignancy rate compared to other RSSs. In our study, both the unnecessary FNA rate and missed malignancy rate were comparable between ACR-TIRADS and C-TIRADS, and both were lower than those observed with ATA guidelines. Specifically, ATA guidelines recommended FNA for 38 benign nodules, of which 20 were classified as TR2 by ACR-TIRADS and TR3 by C-TIRADS, leading to their exclusion from FNA recommendations. These 20 nodules exhibited features such as mixed composition, isoechoic or hyperechoic characteristics, a wider-than-taller shape, a smooth margin, and no calcification. All three RSSs demonstrated a high missed malignancy rate ranging from 37.04% to 50.00% based on their original suggested FNA thresholds. However, our results indicated an overall reduction in the missed malignancy rate (0-8.70%) without affecting the unnecessary FNA rate when using the simulated FNA thresholds. This aligns with the findings of Kim et al. (28). These results suggest that lowering the FNA thresholds for highly suspicious malignant nodules could be an effective strategy for managing malignant thyroid nodules in pediatric patients.

Our study has certain limitations that should be considered. Firstly, the thyroid nodules included in our study were exclusively from our surgical inpatients, potentially introducing a selection bias and limiting the generalizability of our findings to the broader population of thyroid nodules. This skewed sample may have resulted in a higher observed risk of cancer. Secondly, the pathology of some benign nodules obtained through FNA may have produced false-negative results compared to the use of surgical pathology as the gold standard. Thirdly, the non-classification of some nodules could have led to a potential misestimation of the diagnostic performance of ATA guidelines.

## 5 Conclusions

In summary, our study indicates that ACR-TIRADS, C-TIRADS, and ATA guidelines exhibit robust overall diagnostic performance for thyroid nodules in pediatric patients, comparable to that observed in the adult population. All three RSSs showed elevated missed malignancy rates when applying their original recommended FNA criteria. However, when biopsying nodules < 1 cm with the highest category in each RSS, the MMR decreased. Further investigations with larger sample sizes are warranted to validate whether adjusting RSS thresholds is a more appropriate approach for pediatric patients with thyroid nodules.

## Data availability statement

The raw data supporting the conclusions of this article will be made available by the authors, without undue reservation.

## Ethics statement

The research and clinical trials were approved by the ethics committee of The First Affiliated Hospital of Zhengzhou University in China. The studies were conducted in accordance with the local legislation and institutional requirements. The ethics committee/institutional review board waived the requirement of written informed consent for participation from the participants or the participants’ legal guardians/next of kin because Given the retrospective nature of the study, written informed consent was waived as it involves the analysis of existing data. Written informed consent was not obtained from the minor(s)’ legal guardian/next of kin, for the publication of any potentially identifiable images or data included in this article because Given the retrospective nature of the study, written informed consent was waived as it involves the analysis of existing data.

## Author contributions

JY: Conceptualization, Formal analysis, Visualization, Writing – original draft, Writing – review & editing. YC: Formal analysis, Resources, Writing – review & editing. CF: Data curation, Writing – review & editing. XM: Data curation, Writing – review & editing. CS: Methodology, Writing – review & editing. YH: Methodology, Writing – review & editing. KC: Supervision, Writing – review & editing. YZ: Supervision, Writing – review & editing.
